# Lipid Metabolic Dysregulation Driven by *OSMR* Mutations Underlies Amyloidogenesis in Primary Localized Cutaneous Amyloidosis

**DOI:** 10.1002/advs.78068

**Published:** 2026-09-29

**Authors:** Huiting Liu, Wen Zheng, Chao Yang, Tao Yang, Qiuyu Chen, Ping Lu, Huan Yang, Jun Liu, Bin Yang

**Affiliations:** ^1^ Dermatology Hospital Southern Medical University Guangzhou China; ^2^ The First School of Clinical Medicine Southern Medical University Guangzhou China

**Keywords:** AKR1B10, lipid metabolism, OSMR mutation, primary localized cutaneous amyloidosis, protein aggregates, STAT5

## Abstract

**Background**: Primary localized cutaneous amyloidosis (PLCA) is characterized by the deposition of amyloid material within the superficial dermis. Its origin and composition remain debated, including whether deposits derive from keratin filament degeneration or misfolded protein aggregation, and whether non‐protein components contribute.

**Objectives**: This study aimed to re‐examine the composition of PLCA deposits and elucidate how *OSMR* mutations drive their formation, focusing on protein aggregation and lipid metabolism.

**Methods**: We employed PROTEOSTAT staining, lipidomics, and Nile Red/Oil Red O staining in patient tissues, engineered HaCaT cells, and mouse models, together with mechanistic functional analyses.

**Results**: PLCA lesions contain abundant protein aggregates alongside significant lipid accumulation, indicating pathological protein–lipid complexes. As the common genetic defect in familial PLCA, OSMRβ deficiency significantly promoted both lipid accumulation and protein aggregation in cellular and animal models. Lipidomic profiling revealed alterations in unsaturated fatty acids (UFAs, C18:1/C18:2) and ceramides. Crucially, exogenous UFAs induced protein aggregation. Mechanistically, OSMRβ deficiency impairs STAT5 activation, leading to AKR1B10 upregulation and lipid dysregulation, whereas inhibition of this axis rescued both phenotypes.

**Conclusions**: *OSMR* mutation drives amyloid deposition in PLCA through a STAT5–AKR1B10 lipid metabolic axis that promotes co‐aggregation of lipids and misfolded proteins, providing potential therapeutic targets.

## Graphical abstract

*OSMR* mutations disrupt STAT5 signaling and AKR1B10‐mediated lipid metabolism, leading to lipid accumulation and protein aggregation in keratinocytes. This metabolic reprogramming drives amyloid deposition in primary localized cutaneous amyloidosis, revealing a mechanistic link between lipid dysregulation and amyloidogenesis.

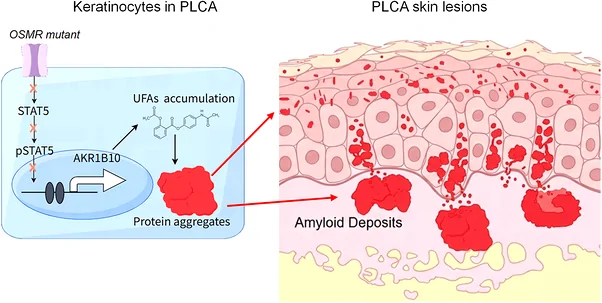

## 1 Introduction

Primary localized cutaneous amyloidosis (PLCA) is a chronic, intensely pruritic skin disorder that is pathologically characterized by the accumulation of amyloid deposits within the superficial dermis [1, 2]. Clinically, PLCA presents as macular amyloidosis (MA) or lichen amyloidosis (LA), characterized by persistent and severe pruritus [3]. For decades, the prevailing hypothesis regarding the origin of these deposits has focused on degenerating keratinocytes. It is postulated that cytokeratins (notably KRT5 and KRT14) [4], along with other proteins such as immunoglobulins [5] and serum amyloid P components (SAP) [6], are released from epidermal cells and form the core of these deposits. This view is strongly supported by immunohistochemical evidence [7] and characteristic apple‐green birefringence under polarized light after Congo red staining [8], which indicates a β‐sheet‐rich structure.

However, this protein‐centric paradigm, while identifying the source of precursor materials, leaves fundamental questions unanswered. Most notably, whether these deposits are best regarded as passive cellular debris or structured aggregates of misfolded proteins. This distinction is central to understanding amyloidogenesis in other diseases. Although lipid‐associated proteins such as apolipoprotein E have been detected in PLCA deposits [9], the potential functional role of lipids themselves remains speculative and largely unexplored.

Genetic studies have revealed that pathogenic mutations in the oncostatin M receptor (*OSMR*) gene, particularly within its extracellular fibronectin type III domains, account for approximately two‐thirds of familial PLCA cases and are correlated with earlier disease manifestation [10, 11]. The *OSMR*‐encoded receptor subunit (OSMRβ) is essential for keratinocyte homeostasis. Our previous study demonstrated that pathogenic *OSMR* mutations (p.G513D and p.P694L) disrupt downstream STAT5 signaling, leading to aberrant keratinocyte differentiation and proliferation [12, 13]. While this genetic link explains keratinocyte dysfunction and the source of amyloid precursor proteins, it does not fully clarify how these materials are transformed into structured amyloid deposits that are characteristic of the disease.

Interestingly, research on systemic and neurodegenerative amyloidoses has significantly expanded our understanding of amyloidogenesis, highlighting the crucial role of lipids in facilitating this process [14, 15, 16]. Currently, lipids are recognized as potent catalysts for protein misfolding and as integral components of amyloid plaques in conditions such as Alzheimer's disease [17, 18]. In an aging study, lipid dysregulation has also been indicated to promote protein aggregation [19]. Notably, OSM/OSMR signaling regulates lipid metabolism by upregulating fatty acid (FA) oxidation enzymes (ACSL3 and CPT1A). OSMRβ deficiency in mice (*Osmr* ^−^ *^/^* ^−^) results in a spontaneous obese phenotype with profound lipid clearance defects [20, 21, 22]. This presents a compelling yet unexplored possibility: *OSMR* mutations drive amyloid deposition in PLCA not only through proteostatic disruption, but also through profound lipid metabolic dysregulation. Could lipids represent previously overlooked constituents of amyloid material?

However, this hypothesis has not yet been tested systematically. The presence of lipids within PLCA lesions and their potential contribution to amyloid formation remain unknown. To address this knowledge gap, we conducted a comprehensive study to characterize the composition of PLCA deposits and elucidate the mechanisms by which *OSMR* mutations drive their formation. By integrating lipidomic and proteomic analyses of patient‐derived samples with mechanistic studies in genetically modified keratinocytes and mouse models, we identified a novel pathway by which *OSMR* mutations induce pathogenic lipid accumulation. This lipid dysregulation promotes the co‐aggregation of lipids and misfolded proteins, fundamentally redefining both the composition of amyloid material and the mechanisms underlying their formation.

## 2 Materials and Methods

### 2.1 Clinical Sample Collection and Processing

Skin biopsies were obtained from patients diagnosed with PLCA at the Dermatology Hospital of the Southern Medical University between June 2020 and September 2024. Diagnosis was confirmed by clinical presentation and histopathological examination, which indicated characteristic Congo red‐positive amyloid deposits under polarized light microscopy. Both lesional and perilesional skin samples were collected from each patient. All cases were carefully evaluated to exclude systemic amyloidosis. Patient demographics and clinical characteristics are summarized in Table S1. Tissues were either immediately processed for experiments or stored at –80°C for later analysis. The study was conducted in accordance with the Declaration of Helsinki, and the protocol was approved by the Institutional Review Board of the Dermatology Hospital of Southern Medical University (Ethics Approval No: 2021069). Written informed consent was obtained from all participants.

### 2.2 Animal Models and Genotyping

Age‐matched wild‐type (WT), *Osmr knockout* (*Osmr^−/−^*), and *Osmr* p.S691L knock‐in mice (*Osmr‐mu*, corresponding to human *OSMR* p.P694L mutation) on a C57BL/6 background were used. The *Osmr‐mu* mouse model was generated through CRISPR‐Cas9‐mediated genome editing to introduce a serine‐to‐leucine substitution at codon 691. Animals were maintained under specific pathogen‐free conditions at 22 ± 2°C and 50% ± 10% humidity, with a 12‐h light/dark cycle. Genotyping was performed by polymerase chain reaction (PCR) amplification of tail deoxyribonucleic acid (DNA), followed by Sanger sequencing to confirm the targeted mutation. All animal experiments were conducted in accordance with the National Institutes of Health Guide for the Care and Use of Laboratory Animals, and the protocol was approved by the Animal Ethics Committee of Southern Medical University (Animal Ethics Approval No: 2025D042).

### 2.3 Cell Culture and Genetic Manipulation

The human immortalized keratinocyte cell line HaCaT was cultured in Dulbecco's Modified Eagle Medium supplemented with 10% heat‐inactivated fetal bovine serum and 1% penicillin‐streptomycin at 37°C in a humidified atmosphere of 5% CO_2_. *OSMR KO* cells [12] were generated using CRISPR‐Cas9 technology, and point mutations (p.G513D and p.P694L) were introduced by site‐directed mutagenesis. For rescue experiments, WT *OSMR* was reintroduced into *KO* cells using lentiviral transduction [12]. All cell lines were regularly tested for mycoplasma contamination and maintained at subconfluent densities during experiments.

### 2.4 Protein Aggregate Detection and Quantification

Cells and tissue sections were fixed with 4% paraformaldehyde for 15 min at room temperature, followed by permeabilization with 0.5% Triton X‐100 in phosphate‐buffered saline (PBS) containing 3 mM ethylenediaminetetraacetic acid (EDTA; pH 8.0) for 30 min. After washing, samples were incubated with PROTEOSTAT Aggresome detection (Enzo Life Science, 1:2000 dilution) and 4′,6‐diamidino‐2‐phenylindole (DAPI) nuclear stain (1 µg/mL) for 30 min in the dark. The PROTEOSTAT dye exhibits fluorescence emission that directly correlates with β‐sheet density, specifically detecting rigid amyloid‐like aggregates [23]. In its unbound state, the dye remains non‐fluorescent due to the free rotation of its aromatic moieties. Upon binding to protein aggregates, the restricted molecular rotation dramatically increases the quantum yield, generating intense fluorescence that enables the sensitive detection of early aggregation states with superior spatiotemporal resolution compared to conventional Congo red staining.

Fluorescence images were obtained using a Leica SP8 confocal microscope with 63× oil immersion objective, exciting at 555 nm for protein aggregates and 360 nm for nuclei. Quantitative analysis was performed on 3–5 randomly selected fields per sample using ImageJ software, with consistent acquisition parameters maintained across all samples.

### 2.5 Lipid Staining and Visualization

Frozen tissue sections from patients with PLCA and normal controls were processed for lipid visualization using Nile Red, BODIPY 493/503, and Oil Red O staining. After air‐drying and PBS washing to remove optimal cutting temperature compound, sections were fixed with 4% paraformaldehyde and permeabilized with 0.5% Triton X‐100 in PBS containing 3 mM EDTA (pH 8.0).

Tissue sections were incubated with Nile Red working solution (1 µg/mL) to detect both polar and nonpolar lipids through their environment‐sensitive fluorescence properties. Parallel samples were stained with BODIPY 493/503 (1 µM) for neutral lipid visualization or Oil Red O (0.3% in 60% isopropanol) to specifically identify esterified FA. All staining procedures were conducted for 30 min at room temperature in the dark, followed by PBS washing and DAPI nuclear counterstaining. Fluorescence images were obtained at 20× and 40× magnification, with lipid content quantified by measuring integrated fluorescence intensity per field.

### 2.6 Lipidomics Analysis

Skin tissues from two‐month‐old WT and *Osmr^−/−^* mice (n = 3 per group), as well as human PLCA samples, were homogenized in ice‐cold methanol. Lipid extraction was performed using methyl‐tert‐butyl ether/methanol/water (10:3:2.5, v/v/v) system. Lipid profiling was conducted on a Waters UPLC‐Q‐TOF‐MS system equipped with a C18 reversed‐phase column. Mass spectra were acquired in both positive and negative ionization modes. Lipid identification was achieved by matching m/z values and retention times against the LipidBlast database. Multivariate statistical analysis, including principal component analysis (PCA) and orthogonal partial least squares discriminant analysis (OPLS‐DA), was performed using MetaboAnalyst software (version 5.0).

### 2.7 Western Blotting

Protein extraction from clinical specimens and cultured cells was performed using radio‐immunoprecipitation assay lysis buffer containing protease and phosphatase inhibitors. Tissue samples were homogenized under liquid nitrogen before lysis. Protein concentrations were determined by bicinchoninic acid assay. Samples were separated by sodium dodecyl sulfate‐polyacrylamide gel electrophoresis and transferred to polyvinylidene fluoride membranes. After blocking, membranes were incubated overnight at 4°C with primary antibodies against AKR1B10 (1:1000) and Tubulin (1:5000), followed by horseradish peroxidase‐conjugated secondary antibodies (1:5000) and electrochemiluminescence detection.

### 2.8 Quantitative Real‐Time PCR

Total RNA was isolated using TRIzol reagent and then reverse‐transcribed according to standard protocols. Quantitative PCR (qPCR) was performed using SYBR Green chemistry with primers indicated in Table S2. Reactions were conducted according to the manufacturer's recommended cycling conditions. All samples were analyzed in triplicate using the 2^–ΔΔCt^ method.

### 2.9 Primary Mouse Keratinocyte Isolation and Culture

Neonatal pups (within three days after birth) from *Osmr^+/−^* intercrosses were euthanized, and skin was dissected. After dispase treatment (2.5 mg/mL) and collagenase IV digestion (1 mg/mL), cell suspensions were filtered through a 70 µm mesh. Following genotyping (primers in Table S2), keratinocytes from WT and *Osmr^−/−^* littermates were cultured in keratinocyte growth medium. Cells were treated with mouse OSM (10 ng/mL) for 48 h before analysis.

### 2.10 AKR1B10 Genetic Manipulation

HaCaT cells were transfected with AKR1B10‐specific small interfering RNA (siRNAs; 100 nM) using RNAiMAX for knockdown experiments. For overexpression, lentiviral vectors containing AKR1B10 complementary DNA were produced in HEK293T cells and used to transduce HaCaT cells. Efficiency was verified by qPCR and Western blotting after 48 h. Nile Red staining was used to assess lipid content.

### 2.11 Luciferase Reporter Assays

The AKR1B10 promoter region (−2000 to +200 bp relative to the transcription start site) was cloned into the pGL3‐basic vector. HaCaT cells were co‐transfected with promoter‐reporter constructs and STAT5 expression vectors using Lipofectamine 3000. After 24 h, cells were treated with either STAT5 inhibitor (5 µM SH‐4‐54) or STAT5 agonist (200 IU/mL interleukin IL‐2) for 12 h to investigate differential regulation of AKR1B10 transcription. Firefly luciferase activity was normalized to Renilla luciferase for control of transfection efficiency.

### 2.12 Statistical Analysis

All experiments were performed with at least three independent biological replicates. Data are presented as mean ± standard deviation (SD). Normality of distribution was assessed using the Shapiro–Wilk test. For comparisons between two groups, Student's t‐test or Welch's t‐test was applied based on variance homogeneity evaluated by Levene's test. Multiple group comparisons were analyzed by one‐way analysis of variance (ANOVA) followed by appropriate post‐hoc tests (least significant difference for equal variances or Dunnett's T3 for unequal variances). Statistical significance was set at *p* < 0.05. All analyses were conducted using the GraphPad Prism software (version 9).

## 3 Results

### 3.1 PLCA Amyloid Deposits are Enriched With Protein Aggregates and Lipids

Our previous proteomic analysis [13] of PLCA amyloid deposits revealed a complex and heterogeneous composition. In addition to cytokeratins (KRT5, KRT14), which are considered the primary components, the deposits were significantly enriched with proteins involved in lipid metabolism and transport (e.g., ApoE, FABP), molecular chaperones (e.g., HSP70, HSP90), and various cytoskeletal proteins (Figure S1A,B). Bioinformatics analysis indicated that these proteins were enriched not only in cytoskeleton‐related functions but also significantly involved in lipid metabolic processes and unfolded protein binding processes (Figure 1A). This result suggests that aberrant lipid metabolism and impaired protein folding may cooperatively contribute to PLCA amyloid formation.

**FIGURE 1:**
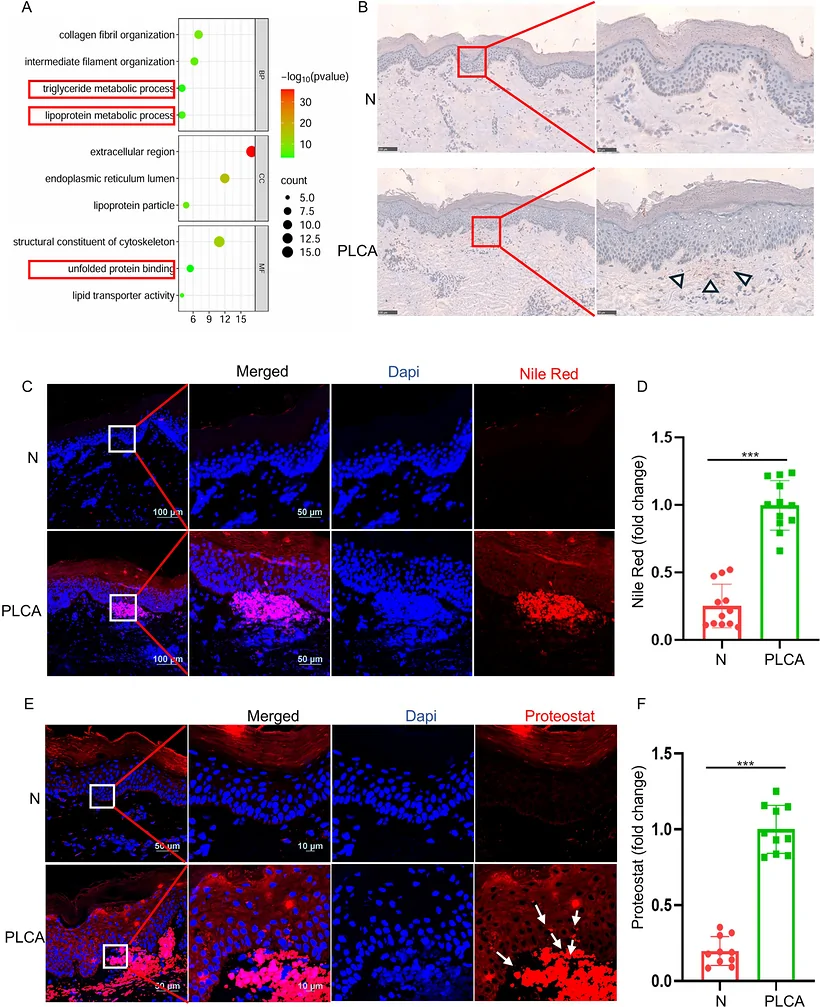
PLCA amyloid deposits represent pathological complexes of protein aggregates and lipids. (A) Functional enrichment analysis (GO terms) of proteins identified by mass spectrometry in Congo red‐positive amyloid material microdissected from PLCA patient lesions. The analysis highlights involvement in cytoskeleton organization, lipid metabolic processes, and unfolded protein binding. (B) Congo Red staining results of patients with PLCA compared to normal (N) skin. (scale bars: 200 and 50 µm). (C) Immunofluorescence results of Nile Red staining in skin tissues from healthy controls and PLCA patients with OSMR mutations (n = 3, PLCA = 3) (scale bars: 50 and 10 µm). (D) Quantification of Nile Red fluorescence intensity shown in (C), normalized to cell number (*p < 0.05, one‐way ANOVA). (E) Immunofluorescence results of PROTEOSTAT protein aggregation assay in skin tissues from healthy controls and PLCA patients with *OSMR* mutations (n = 3, PLCA = 3) (scale bars: 50 and 10 µm). (F) Quantification of PROTEOSTAT fluorescence intensity shown in (E), normalized to cell number (*p < 0.05, one‐way ANOVA).

Prompted by the proteomic findings, we subsequently performed histological assessments of lipid content and protein aggregation status in patient tissues. Lipid staining on skin samples from PLCA patients harboring *OSMR* mutations revealed significant lipid accumulation within the amyloid deposit regions (Figure 1B‐D, Figure S1C,D). Consecutively, using PROTEOSTAT staining, specific for misfolded protein aggregates, we detected widespread protein aggregation signals within keratinocytes and at the sites of amyloid deposition in the same pathological areas (Figure 1E,F). High‐resolution imaging revealed distinct morphological patterns, including fine granular aggregates distributed within the keratinocyte cytoplasm and larger, brighter accumulations at cell‐cell junctions (Figure 1E, arrows). The dermal papillae contained substantial aggregated deposits that appeared continuous with smaller aggregates along epidermal migratory pathways. This co‐localization of lipids and protein aggregates indicates that PLCA amyloid deposits are complex pathological composites containing misfolded protein aggregates and lipids.

### 3.2 OSMRβ Deficiency Recapitulates the Lipid and Protein Aggregation Phenotype of PLCA in Mouse Epidermis and Human Keratinocytes

Given that *OSMR* mutations are a primary genetic cause of familial PLCA, we next investigated whether OSMRβ deficiency is responsible for the observed lipid and protein aggregation. To determine if these changes resulted directly from OSMRβ dysfunction rather than secondary disease effects, we analyzed skin from *Osmr KO* (*Osmr^−/−^*) and Osmr p.S691L missense mutant (*Osmr‐mu*) mouse models (Figure 2). Compared to wild‐type (WT) mice exhibiting minimal signals, mice with *Osmr KO* and *Osmr* mutations developed diffuse lipid accumulation (Figure 2A,B) and prominent protein aggregates (Figure 2C,D) throughout their epidermis. The similarity between human and mouse phenotypes strongly suggests that *OSMR* mutations intrinsically disrupt both lipid homeostasis and protein folding in keratinocytes.

**FIGURE 2:**
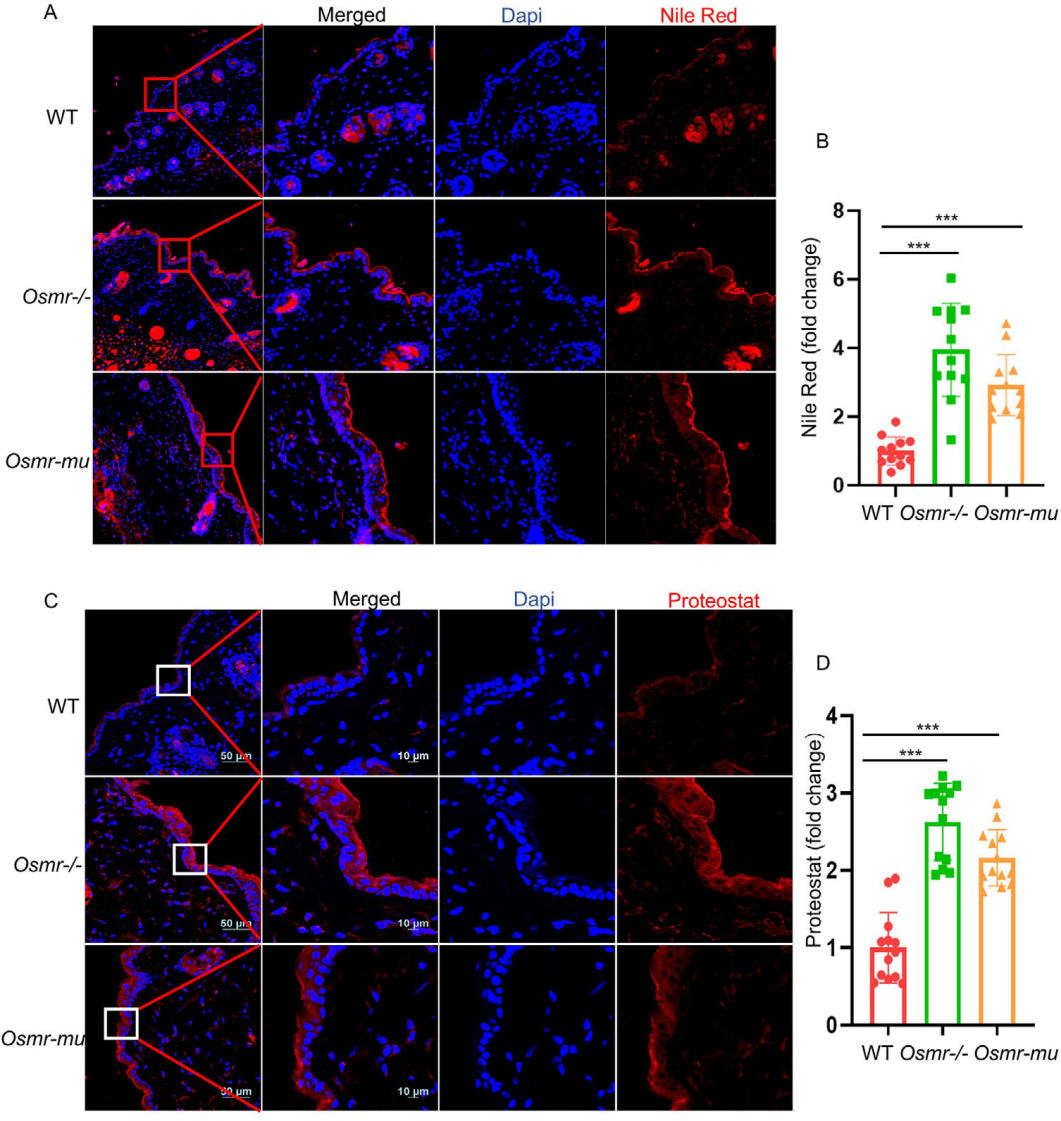
OSMRβ deficiency induces lipid accumulation and protein aggregation in mice. (A) Immunofluorescence of Nile Red staining in skin tissues of WT, *Osmr^−/−^*, and *Osmr‐mu* mice (n = 3 per group) (scale bars: 50 µm and 10 µm). (B) Quantification of normalized Nile Red fluorescence intensity shown in (A), normalized to cell number (*p < 0.05, one‐way ANOVA). (C) Immunofluorescence results of PROTEOSTAT protein aggregation assay in the epidermis of WT, *Osmr^–/–^*, and *Osmr* p691 mutation mice (n = 3, n = 3, and n = 3) (scale bars: 50 and 10 µm). (D) Quantification of normalized PROTEOSTAT fluorescence intensity shown in (C), normalized to cell number (*p < 0.05, one‐way ANOVA).Data are presented as mean ± SD. n.s., no statistical difference; *p < 0.05, **p < 0.01. WT: wild‐type; *Osmr^–/–^*: *Osmr* knockout mice; *Osmr‐mu*: *Osmr p.S691L* mutant mice.

To directly test whether *OSMR* mutations drive protein aggregation, we established immortalized human keratinocyte (HaCaT) models with either complete *OSMR KO* or specific point mutations (p.G513D and p.P694L) [12]. Both OSMRβ‐deficient models developed elevated basal lipid levels (Figure 3A, left; Figure S6A), and prominent perinuclear protein aggregates (Figure 3B,E), which were completely absent in WT cells.

**FIGURE 3:**
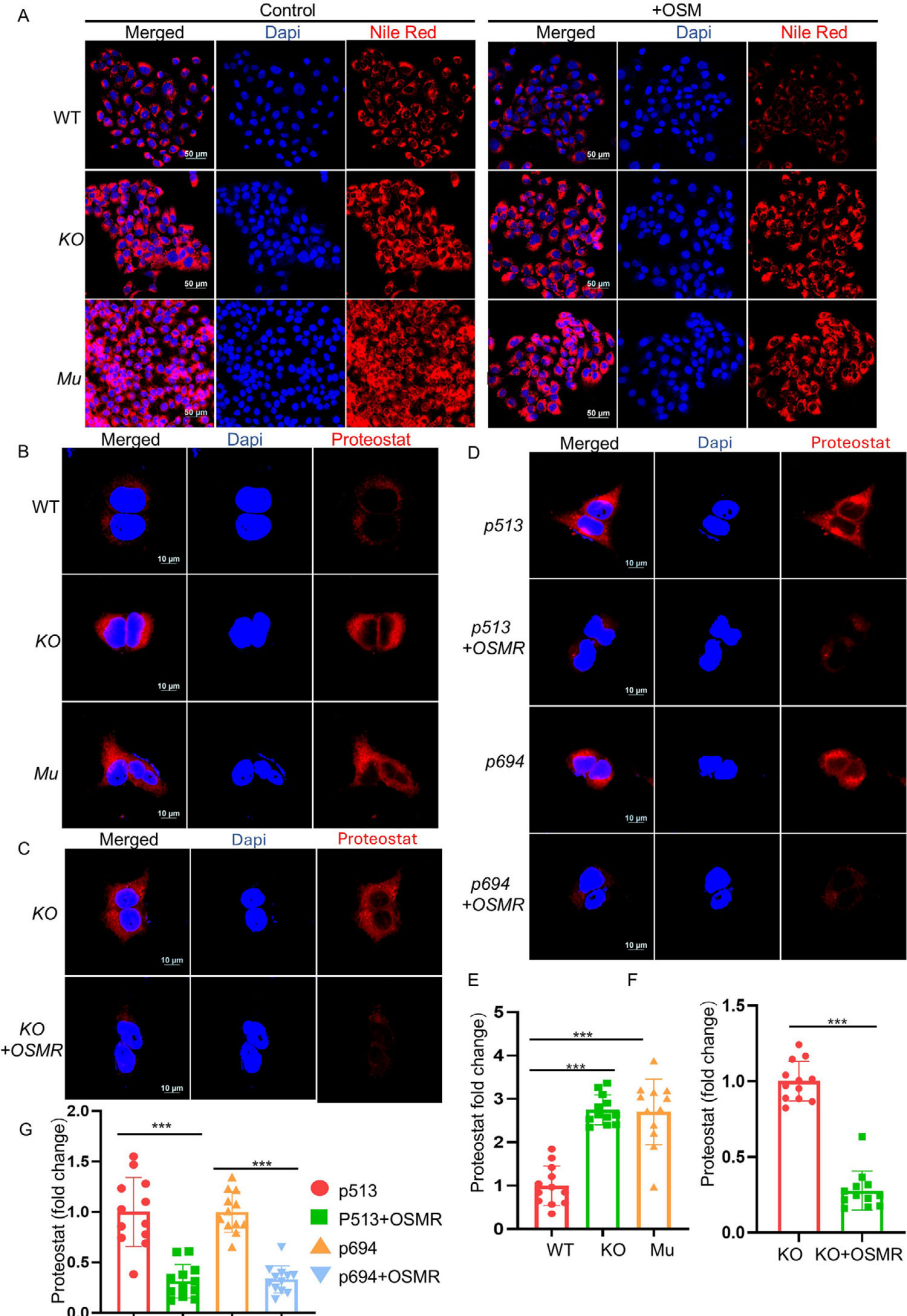
OSMRβ deficiency drive lipid accumulation and protein aggregation in keratinocytes. (A) Immunofluorescence results of Nile Red staining in in WT, *OSMR‐KO*, and *OSMR‐mutant* HaCaT cells with/without OSM treatment (scale bar: 50 µm). (B) Immunofluorescence results of PROTEOSTAT protein aggregation assay in HaCaT cells with *OSMR KO* and mutation (scale bars: 10 µm). (C) Immunofluorescence results of PROTEOSTAT protein aggregation assay in *OSMR KO* HaCaT cells after reconstitution with WT *OSMR* (scale bar: 10 µm). (D) Immunofluorescence results of PROTEOSTAT protein aggregation assay in *OSMR* mutant HaCaT cells after reconstitution with WT *OSMR* (scale bar: 10 µm). (E–G) Quantitative analysis of PROTEOSTAT fluorescence intensity normalized per cell for conditions indicated in B, C, and D, respectively. Data represent mean ± SD (n ≥ 3 independent experiments). Statistical significance was determined by one‐way ANOVA (*p < 0.05, **p < 0.01; n.s., not significant). WT: wild‐type; *KO*: *OSMR* knockout HaCaT cells; *Mu*: *OSMR* mutant HaCaT cells. *Mu* cells include p.G513D and p.P694L mutations generated by site‐directed mutagenesis. *KO+OSMR*, *p513+WT OSMR*, and *p694+WT OSMR* indicate reconstitution of wild‐type *OSMR* in knockout or mutant cells, respectively.

To further confirm the specific role of OSMRβ signaling in regulating keratinocyte lipid homeostasis, we performed ligand stimulation experiments with OSM. In wild‐type HaCaT cells, OSM stimulation significantly reduced intracellular lipid levels (Figure 3A, right), indicating a negative regulatory role of OSMRβ signaling in lipid homeostasis. In contrast, in both *OSMR* knockout cells and HaCaT cells carrying the p.G513D/p.P694L mutations, OSM stimulation failed to induce a similar reduction in lipid content. These cells maintained elevated basal lipid levels regardless of OSM treatment (Figure 3A, right). This complete loss of responsiveness observed in genetically modified cells provides definitive evidence that the regulation of keratinocyte lipid homeostasis by OSM is exclusively mediated through functional OSMRβ signaling.

Critically, reintroduction of WT *OSMR* into the mutant cells completely rescued both the protein aggregation (Figure 3C,D) and lipid accumulation phenotypes. These data collectively establish that proper OSMRβ function is essential for maintaining both lipid and protein homeostasis in keratinocytes.

### 3.3 *OSMR* Mutation Induce Accumulation of Unsaturated Fatty Acids in Amyloid Deposits

Emerging evidence suggests that lipid dysregulation contributes to amyloid formation in multiple disorders, including Alzheimer's disease (Aβ) and Parkinson's disease (α‐Syn) [24, 25]. Previous studies have demonstrated that OSM signaling through OSMRβ upregulates FA oxidation enzymes such as ACSL3 and CPT1A, thereby promoting lipid clearance [20]. The metabolic disturbances observed in *Osmr^−/−^* mice, including spontaneous obesity and lipid accumulation [21, 22], further support this regulatory role. Based on these findings, we hypothesized that *OSMR* mutations impair OSM‐mediated lipid homeostasis in keratinocytes, thereby facilitating protein aggregation in PLCA.

Through comprehensive, untargeted lipidomics profiling, we characterized the lipid profiles of skin samples from patients with PLCA who had *OSMR* mutations, comparing lesional, perilesional, and normal control tissues. This analysis identified 1,261 distinct lipid species (Figure S2A). The detected lipids represented 11 major classes, including triglycerides (TG), ceramides (Cer), free FA, sphingomyelins (SM), and various phospholipid species (PE, PC, PS, LPE, and LPC). The PCA of the global lipid profiles revealed progressive metabolic alterations across sample types. While lesional and normal skin samples exhibited clear separation along the first principal component, perilesional tissues consistently occupied an intermediate position within this metabolic continuum (Figure 4A; Figure S2B–E). This distinct spatial distribution suggests that perilesional regions represent a transitional metabolic state during PLCA progression, potentially reflecting ongoing pathological remodeling of lipid homeostasis in keratinocytes at the lesion periphery.

**FIGURE 4:**
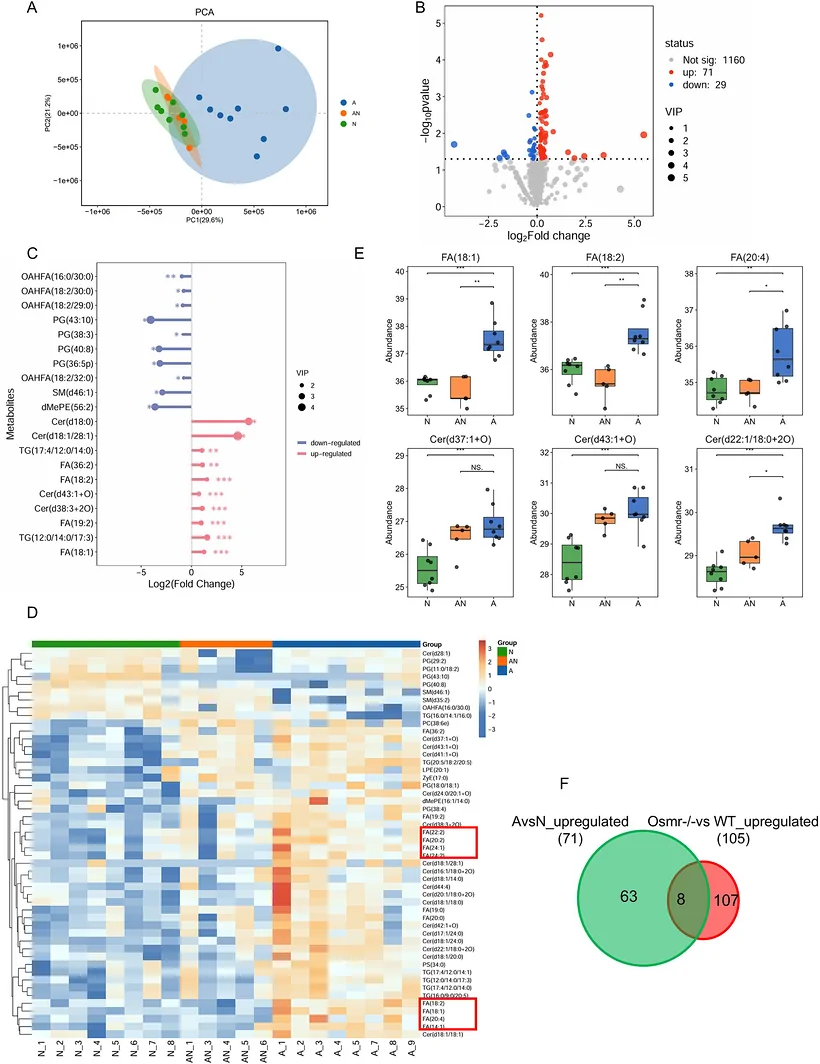
Lipidomics Reveals *OSMR* Mutation induced Accumulation of UFAs. (A) PCA of lipidomic data from PLCA lesions, adjacent skin, and normal skin. (B) Volcano plot of lipidomic data comparing PLCA lesions to normal skin. (C) Stick plot of lipidomic data comparing PLCA lesions to normal skin. (D) Heatmap of differential lipids between PLCA lesions, normal skin, and adjacent skin. (E) Box plot of differential lipids between PLCA lesions, normal skin, and adjacent skin. (F) Venn diagram intersection of lipid species significantly increased in both PLCA lesions and *Osmr^−/−^* mice skin. Abbreviations: A, lesional skin from PLCA patients; AN, non‐lesional (adjacent) skin from PLCA patients; N, normal skin from healthy donors.

Comparative analysis (Table S3) was used to identify 100 significantly altered lipid species in PLCA lesions (71 upregulated, 29 downregulated; VIP > 1, *p* < 0.05), representing 8.1% of detected lipids (Figure 4B). FA and Cer emerged as the most prominently enriched categories (Figure 4C). Heatmap visualization revealed consistent overexpression patterns for unsaturated FA (UFAs) and Cer in lesional tissue, with perilesional areas indicating intermediate expression (Figure 4D). Notably, lesional samples exhibited considerable variability in UFA and ceramide levels (Figure 4E), reflecting metabolic heterogeneity.

Parallel studies in *Osmr^−/−^* mice recapitulated key aspects of the human lipid profile (Figure S3A–E). Among 2,143 detected lipid species, 212 indicated significant alterations in knockout animals, with particular enrichment of UFAs and TG (Table S4). Intersection analysis was used to identify eight lipid species consistently increased in both human PLCA lesions and *Osmr^−/−^* mice skin, predominantly UFAs and Cer (Figure 4F). These conserved metabolic changes across species suggest that OSMRβ deficiency fundamentally alters keratinocyte lipid metabolism, with the accumulation of UFA representing a shared feature between human PLCA and mouse models. The observed lipid perturbations may create conditions favorable for protein misfolding and aggregation in keratinocytes.

### 3.4 Unsaturated Fatty Acids Promote Pathogenic Protein Aggregation in PLCA Keratinocytes

Based on the consistent identification of increased UFAs in human and mouse models, we hypothesized that these lipids might directly promote protein aggregation. Treatment of HaCaT cells with oleic acid (OA, C18:1) or linoleic acid (LA, C18:2) induced dose‐dependent intracellular lipid accumulation (Figure 5A,B). Importantly, parallel protein aggregate staining revealed that OA and LA significantly induced abnormal protein aggregation, with the strongest proteostat signals in the OA/LA co‐treatment group (Figure 5C,D). These results demonstrate that UFAs, which accumulate due to *OSMR* mutations, promote protein misfolding and aggregation in keratinocytes.

**FIGURE 5:**
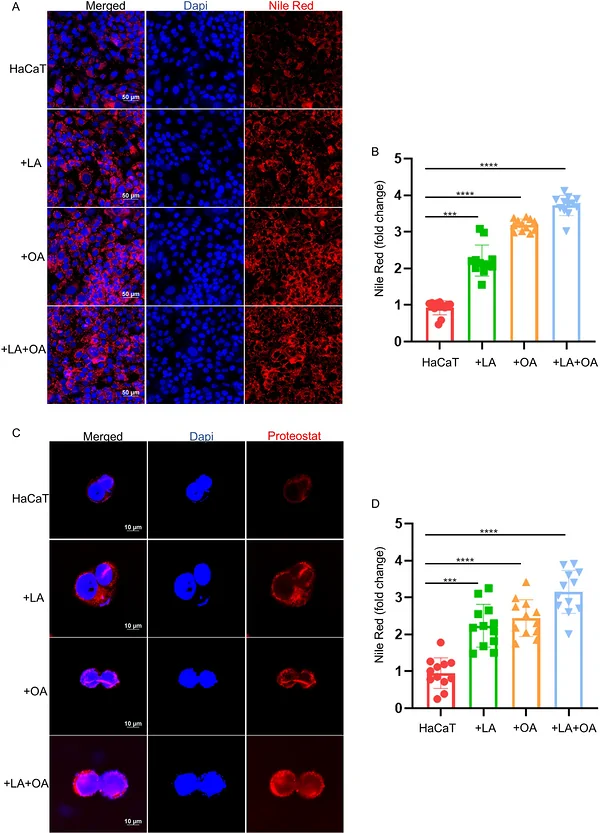
UFAs Promote Protein Aggregation in keratinocytes. (A) Immunofluorescence results of Nile Red staining in HaCaT cells treated with oleic acid and linoleic acid (scale bar: 50 µm). (B) Quantification of normalized Nile Red fluorescence intensity shown in (A), normalized to cell number (*p < 0.05, one‐way ANOVA). (C) Immunofluorescence results of PROTEOSTAT protein aggregation assay in HaCaT cells treated with oleic acid and linoleic acid (scale bar: 10 µm). (D) Quantification of normalized PROTEOSTAT fluorescence intensity shown in (C), normalized to cell number (*p < 0.05, one‐way ANOVA).

### 3.5 *OSMR* Mutations Drive Lipid Accumulation in PLCA via STAT5/AKR1B10 Axis

To elucidate the mechanism linking *OSMR* mutations to UFA accumulation, we analyzed transcriptomic data from OSM‐stimulated HaCaT cells and patient samples with PLCA. This revealed an inverse relationship between OSM signaling and AKR1B10 expression: while OSM treatment downregulated this aldo‐keto reductase, AKR1B10 was markedly upregulated in PLCA lesions. Given previous reports linking AKR1B10 knockdown to reduced lipid levels [25, 26], we confirmed its overexpression in PLCA through multiple approaches. Quantitative PCR and Western blotting analysis revealed elevated AKR1B10 expression in patient samples (Figure 6A,C), whereas immunohistochemistry demonstrated strong staining throughout the PLCA epidermis and amyloid deposits, contrasting with the weak expression observed in normal skin (Figure 6B).

**FIGURE 6:**
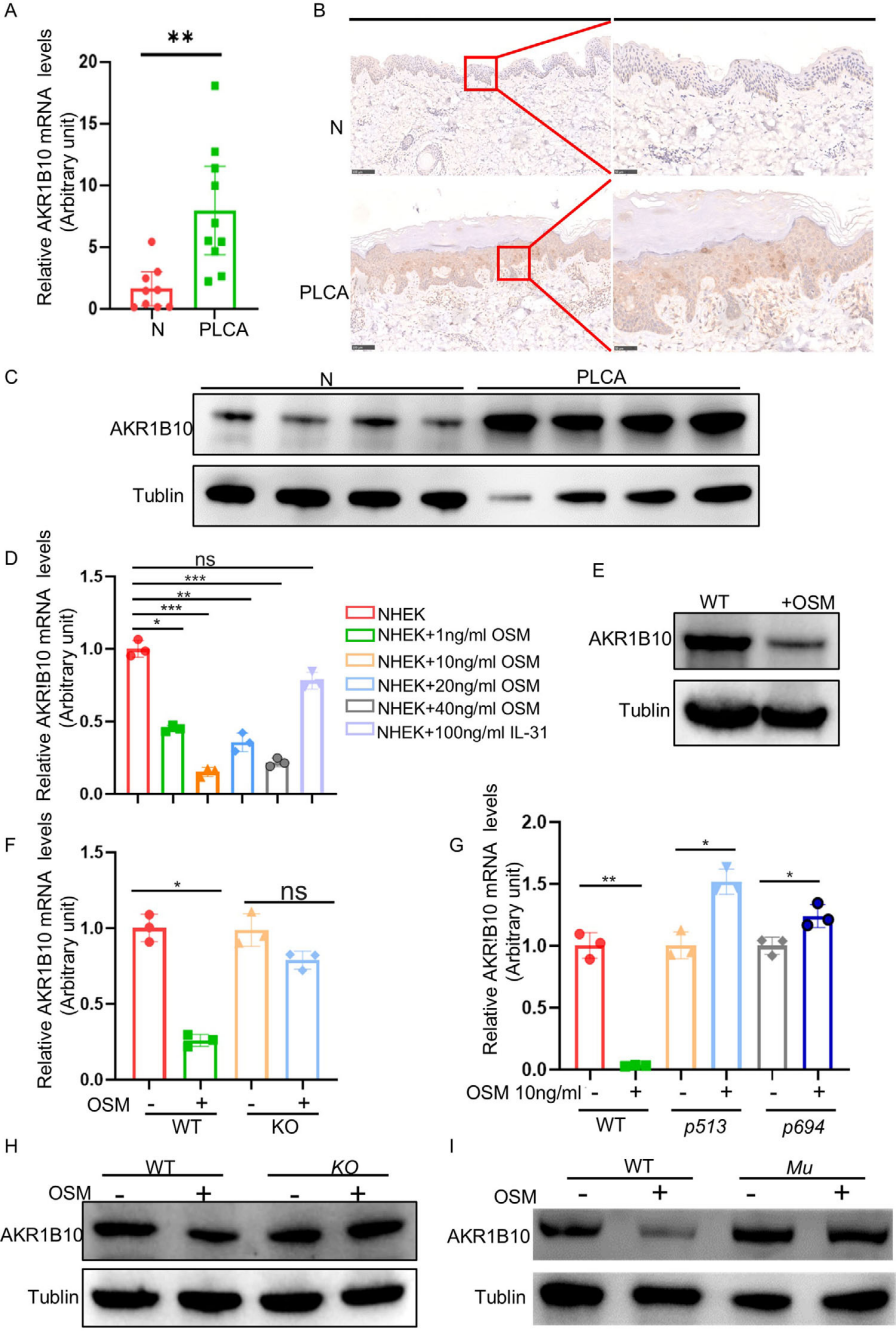
*OSMR* Mutations Upregulate AKR1B10 Through Impaired OSM Signaling. (A) qPCR analysis indicating the difference in AKR1B10 mRNA expression levels between patients with normal skin and PLCA skin (n = 9, n = 9). (B) Immunohistochemical staining indicating the difference in AKR1B10 expression in the epidermis between patients with normal skin and PLCA skin. (C) Western blotting indicating the difference in AKR1B10 protein expression levels between patients with normal skin and PLCA skin. (D) Changes in AKR1B10 mRNA expression levels in HaCaT cells stimulated with different concentrations of OSM and IL‐31. (E) Changes in AKR1B10 protein expression levels in HaCaT cells stimulated with OSM. (F‐G) Changes in AKR1B10 mRNA and protein expression levels in HaCaT cells and *OSMR*‐*KO* HaCaT cells stimulated with OSM. (H‐I) Changes in AKR1B10 mRNA and protein expression levels in HaCaT cells and *OSMR*‐mutant HaCaT cells stimulated with OSM.

As OSMRβ serves as a receptor subunit for both OSM and IL‐31 [10, 27], we compared their effects on AKR1B10 regulation. Graded OSM concentrations produced dose‐dependent suppression of AKR1B10 at mRNA (Figure 6D) and protein levels (Figure 6E), whereas IL‐31 treatment indicated a non‐significant effect, demonstrating ligand specificity in this regulatory pathway. Genetic models provided definitive evidence that OSMRβ is required for OSM to regulate AKR1B10. *OSMR*‐*KO* and *OSMR*‐mutant HaCaT cells no longer responded when OSM was used to inhibit AKR1B10 (Figure 6F–I). Primary keratinocytes isolated from *Osmr*‐*KO* mice (Figure S5A,B) exhibited the same loss of responsiveness, with murine OSM failing to suppress Akr1b10 expression (Figure S5C,D), confirming conservation across species. These parallel results in human and mouse systems demonstrate that this regulatory mechanism is conserved across species.

Bioinformatic analysis predicted STAT5 binding sites in the AKR1B10 promoter. Using the STAT5 inhibitor SH‐4‐54, we found it blocked OSM‐mediated suppression of AKR1B10 expression (Figure 7A,B) and reversed OSM's lipid‐reducing effects (Figure 7C,D). Luciferase reporter assays confirmed that STAT5 activation represses AKR1B10 promoter activity (Figure 7E,F). These results demonstrate that *OSMR* mutations impair STAT5 activation, leading to derepression of AKR1B10 transcription and subsequent lipid dysregulation.

**FIGURE 7:**
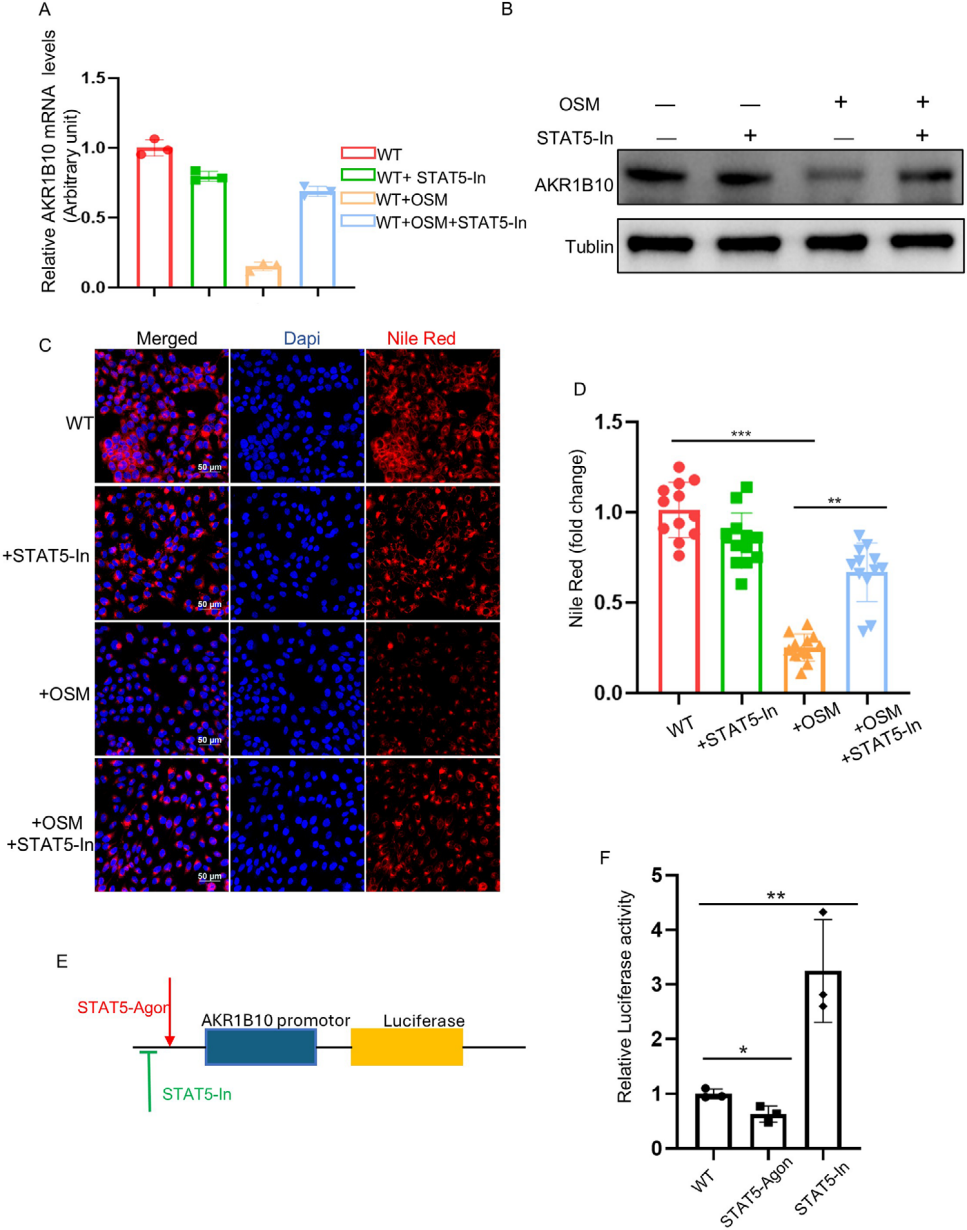
*OSMR* Mutations impair STAT5‐mediated repression of AKR1B10, leading to its upregulation in PLCA. (A‐B) Changes in AKR1B10 mRNA and protein levels after treatment with OSM and STAT5 inhibitor. (C) Nile Red staining results of HaCaT cells after treatment with OSM and STAT5 inhibitor. (D) Quantification of normalized Nile Red fluorescence intensity shown in (C), normalized to cell number (*p < 0.05, one‐way ANOVA). (E) Schematic diagram of the luciferase assay to detect the binding of STAT5 to the AKR1B10 promoter. (F) Changes in luciferase activity after treatment with STAT5 inhibitor and agonist.

### 3.6 AKR1B10 Mediates Lipid‐Driven Protein Aggregation Downstream of *OSMR* Mutations in PLCA

We performed complementary genetic manipulation experiments to establish the functional consequences of AKR1B10 dysregulation. The siRNA‐mediated knockdown of AKR1B10 expression was reduced (Figure 8A,B), accompanied by a decrease in intracellular lipid content (Figure 8C and Figure S6B). Conversely, lentiviral‐mediated overexpression increased AKR1B10 levels (Figure 8D,E), resulting in enlarged lipid droplets with a greater diameter compared to controls (Figure 8F and Figure S6C). Protein aggregate staining in these genetically modified cells revealed a direct correlation between AKR1B10 expression levels and protein aggregation. Cells with AKR1B10 knockdown indicated reduced protein aggregates, while AKR1B10‐overexpressing cells exhibited increased aggregate formation (Figure 8G and Figure S6D). These results demonstrate that AKR1B10 regulates lipid metabolism and influences protein homeostasis in keratinocytes.

**FIGURE 8:**
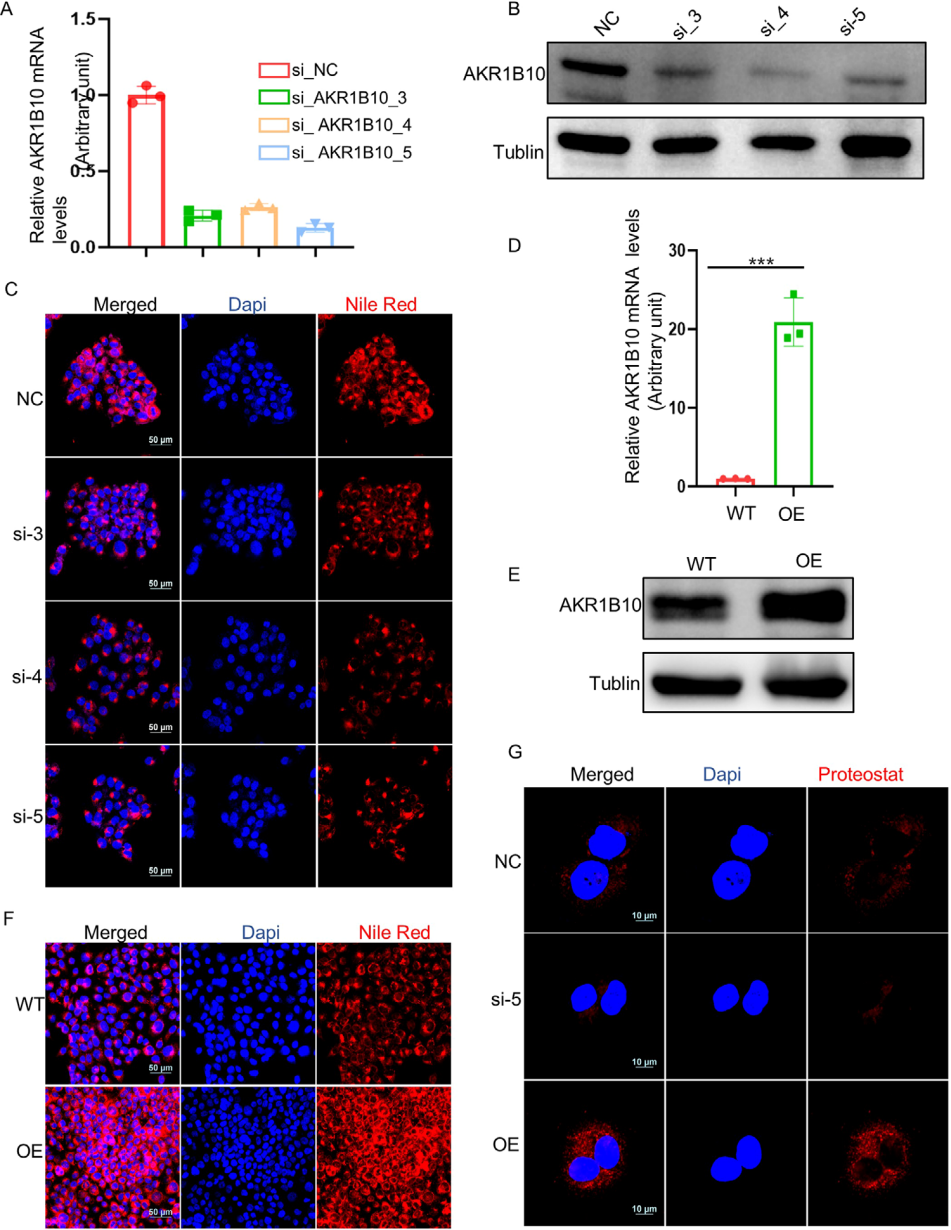
AKR1B10 mediates lipid accumulation and protein aggregation in the pathogenesis of PLCA. (A, B) Changes in mRNA and protein levels of AKR1B10 after knockdown using siRNA in HaCaT cells. (C) Nile Red staining indicating changes in intracellular lipid content after AKR1B10 knockdown. (D, E) Changes in mRNA and protein levels of AKR1B10 after overexpression using viral vectors in HaCaT cells. (F) Nile Red staining indicating changes in intracellular lipid content after AKR1B10 overexpression. (G) Immunofluorescence results of PROTEOSTAT protein aggregation assay in AKR1B10‐knockdown and AKR1B10‐overexpressing HaCaT cells (scale bar: 10 µm).

This provides compelling evidence that AKR1B10 serves as an essential regulator of keratinocyte lipid metabolism downstream of OSMR/STAT5 signaling, and that AKR1B10‐mediated lipid dysregulation directly contributes to proteostasis impairment in PLCA pathogenesis.

## 4 Discussion

For decades, PLCA pathogenesis has been interpreted through the lens of the “cytokeratin debris” hypothesis, which regarded amyloid deposits as passive accumulations of keratin filaments shed from keratinocytes. Our integrated multi‐omics analyses fundamentally challenge this paradigm by demonstrating that PLCA amyloid deposits constitute pathogenic co‐aggregation of misfolded proteins and lipids, driven by active cellular processes under genetic mutations.

First, our study provides definitive evidence regarding the nature and origin of protein aggregation in PLCA, establishing it as an active cellular pathological process. The cornerstone of our findings is the demonstration that these amyloid deposits represent active protein aggregation rather than passive cellular debris. Using PROTEOSTAT staining, a sensitive method specific for β‐sheet‐rich protein aggregates, we identified abundant intracellular protein aggregates within keratinocytes of both patients with PLCA and *Osmr‐mutant* mice. This intracellular localization is significant as it refutes the classical notion of purely extracellular debris accumulation and instead positions protein misfolding as an early and keratinocyte‐autonomous event in disease pathogenesis. The consistency of this finding across human and mouse tissues, and its recapitulation in genetically engineered OSMRβ‐deficient keratinocytes, strongly reinforces this conclusion. Critically, the complete reversal of this aggregation phenotype upon reintroduction of wild‐type OSMR establishes a direct causal link between OSMRβ dysfunction and proteostasis disruption. Mass spectrometry of microdissected amyloid material further revealed a complex composition, enriched not only in keratins but also in apolipoproteins (e.g., ApoE) and molecular chaperones (e.g., HSP70/90), indicating a concomitant and broad failure of cellular protein quality control. The gradient distribution pattern we observed—ranging from small particles (1–3 µm) in the stratum corneum to larger conglomerates (> 20 µm) in the dermis—supports a plausible mechanistic basis for the historical observation of extracellular amyloid deposits.

Second, we identified and mechanistically dissected a central, causative role for lipid metabolic dysregulation in driving amyloidogenesis. Lipid metabolic dysregulation represents another important pathway through which OSMRβ dysfunction influence protein aggregation. Beyond the observed accumulation of UFAs and ceramides, the presence of lipid metabolism‐associated proteins (e.g., ApoE, FABP) within the deposits suggests a profound interplay between lipid dyshomeostasis and protein aggregation. We speculate that these lipid‐rich microenvironments and specific lipid‐protein interactions, potentially acting as “molecular glue,” could facilitate the co‐aggregation of diverse proteins [28, 29], contributing to the unique multi‐component nature of PLCA deposits compared to other amyloidoses. Lipidomic analysis revealed significant increases in unsaturated fatty acids (e.g., oleic acid, linoleic acid) and long‐chain ceramides in PLCA lesions, a pattern that was validated in *Osmr^−/−^* mouse models. Particularly noteworthy are the species‐specific differences in triglyceride metabolism between humans and mice, reflecting evolutionary divergence in epidermal lipid synthesis pathways. In vitro experiments confirmed that UFAs can directly promote protein aggregation, likely through hydrophobic interactions affecting protein conformational stability, similar to lipid‐mediated protein aggregation observed in neurodegenerative diseases [30].

Finally, our findings integrate these pathways into a novel, unified pathogenic paradigm for PLCA. We propose that *OSMR* mutations act as the genetic trigger that concurrently disrupts two key homeostatic pillars in keratinocytes: STAT5‐mediated signaling for lipid metabolism and the machinery for protein folding. The resulting lipid metabolic reprogramming (via AKR1B10/UFA) and proteostatic stress converge at the level of protein‐lipid interactions to drive the formation of complex, mixed co‐aggregates. This “lipid‐protein co‐aggregation” model moves beyond the simplistic debris theory and aligns PLCA with emerging paradigms in systemic amyloidoses and neurodegenerative diseases, where lipid dysregulation is increasingly recognized as a critical facilitator of protein aggregation. Our work thus redefines the essential nature of cutaneous amyloids.

Several limitations of this study should be addressed. The precise molecular pathway of protein aggregate formation in vivo, direct mechanistic evidence of how specific lipid molecules influence protein conformation, and the development of more accurate disease animal models remain important questions for future research. Addressing these issues will further refine our understanding of PLCA pathogenesis.

In conclusion, this study systematically elucidates the molecular mechanism by which *OSMR* mutations drive protein aggregation through lipid metabolic reprogramming, leading to cutaneous amyloidosis in PLCA. For the first time in PLCA research, we established a ternary causal chain of “genetic mutation‐lipid metabolic abnormality‐amyloid deposition,” thereby filling a critical knowledge gap in understanding the disease's pathogenesis.

## Author Contributions

**Huiting Liu**: data curation, investigation, writing – original draft, writing – review and editing, conceptualization, methodology, software, validation, formal analysis, visualization, project administration, resources. **Wen Zheng**: methodology, investigation. **Chao Yang**: resources, funding acquisition. **Tao Yang**: methodology. **Qiuyu Chen**: methodology. **Ping Lu**: resources. **Huan Yang**: resources. **Jun Liu**: writing – review and editing, supervision, resources. **Bin Yang**: project administration, funding acquisition, writing – review and editing, resources.

## Conflicts of Interest

The authors declare no conflicts of interest.

## Supporting information




**Supporting File 1**: advs78068‐sup‐0001‐SuppMat.docx.

**Supporting File 2**: advs78068‐sup‐0002‐S1.jpg.

**Supporting File 3**: advs78068‐sup‐0003‐S2.JPG.

**Supporting File 4**: advs78068‐sup‐0004‐S3.JPG.

**Supporting File 5**: advs78068‐sup‐0005‐S4.JPG.

**Supporting File 6**: advs78068‐sup‐0006‐S5.JPG.

**Supporting File 7**: advs78068‐sup‐0001‐S6.JPG.

**Supporting File 8**: advs78068‐sup‐0008‐S1.xlsx.

**Supporting File 9**: advs78068‐sup‐0009‐S2.xlsx.

**Supporting File 10**: advs78068‐sup‐0010‐S3.xlsx.

**Supporting File 11**: advs78068‐sup‐0011‐S4.xlsx.

## Data Availability

The RNA sequencing data analyzed in this study are available in the Gene Expression Omnibus (GEO) under accession number GSE150994. The lipidomics dataset is available as supplementary table S3 and S4.
