# Supplementary material for: Lipid Metabolic Dysregulation Driven by OSMR Mutations Underlies Amyloidogenesis in Primary Localized Cutaneous Amyloidosis
Source: Adv Sci (Weinh). 2026 Sep 29:e78068. Online ahead of print. doi: 10.1002/advs.78068 (PMC13624327; doi:10.1002/advs.78068)
Supplement: Supplementary file 1 [file ADVS-9999-e78068-s003.docx]

**Supplementary Figure legends**

Supplementary Figure S1. Mass spectrometry results of amyloid substances in PLCA.

Patients with hypersensitivity vasculitis.

(A) Congo Red staining results of patients with PLCA (scale bars: 200 and 50 µm). (B) Proportional composition of amyloid substances.

(C) Immunofluorescence results of BODIPY 493/503 staining in skin tissues from healthy controls and PLCA patients with OSMR mutations (n = 3, PLCA = 3) (scale bars: 50 and 10 µm).

(D) Quantification of normalized BODIPY fluorescence intensity shown in (C), normalized to cell number (*p < 0.05, one-way ANOVA).

Supplementary Figure S2. Differences in lipid profiles between lesions and adjacent/normal skin in patients with PLCA.

(A) Subclassification of lipids and corresponding quantities in lesions, adjacent skin, and normal skin of patients with PLCA.

(B) OPLS-DA of lipidomic data from PLCA lesions and normal skin.

(C) Permutation results for the OPLS-DA model testing of lipidomic data from PLCA lesions and normal skin.

(D) OPLS-DA of lipidomic data from PLCA lesions and adjacent skin.

(E) Permutation results for the OPLS-DA model testing of lipidomic data from PLCA lesions and adjacent skin.

(F) Volcano plot of lipidomic data comparing PLCA lesions to adjacent skin.

(G) Stick plot of lipidomic data comparing PLCA lesions to adjacent skin.

Supplementary Figure S3. Increased levels of FA and TG in Osmr−/− mice compared to WT mice.

(A) OPLS-DA of lipidomic data from Osmr−/− and WT mice skin.

(B) Volcano plot of lipidomic data comparing Osmr−/− and WT mice skin.

(C) Stick plot of lipidomic data comparing Osmr−/− and WT mice skin.

(D) Heatmap of differential lipids between Osmr−/− and WT mice skin.

(E) Box plot of differential lipids between Osmr−/− and WT mice skin.

Supplementary Figure S4. Lipid accumulation in OSMRβ-deficient mice epidermis.

(A) Oil Red O staining of skin tissues from WT, Osmr−/−, and Osmr-mu mice (n = 3 per group) (scale bars: 100 and 50 µm).

Supplementary Figure S5. Osm/Osmr inhibits AKR1B10 expression.

(A) Procedure for isolating primary keratinocytes from neonatal mice.

(B) Genotyping of neonatal mice by extracting DNA.

(C-D) Changes in AKR1B10 mRNA and protein expression levels in keratinocytes derived from WT and Osmr−/− mice stimulated with mOSM.

Supplementary Figure S6. Quantification of lipid accumulation and protein aggregation in HaCaT cells.

(A) Quantification of Nile Red fluorescence intensity in WT, OSMR-KO, and OSMR-mutant HaCaT cells with/without OSM treatment shown in Figure 3A.

(B) Quantification of the Nile Red fluorescence intensity in AKR1B10-knockdown HaCaT cells shown in Figure 8C.

(C) Quantification of the Nile Red fluorescence intensity in AKR1B10-overexpressing HaCaT cells shown in Figure 8F.

(D) Quantification of the PROTEOSTAT fluorescence intensity in AKR1B10-knockdown and AKR1B10-overexpressing HaCaT cells shown in Figure 8G.

Supplementary Table S1. Clinical specimen profiles of patients with PLCA.

Supplementary Table S2. Primer sequences used in this study.

Supplementary Table S3. Differentially expressed lipids in PLCA lesions versus normal skin.

Supplementary Table S4. Differentially expressed lipids in Osmr−/− versus WT mice skin.
